# Comparison of the 4-in-1 block technique with femoral and adductor canal blocks in arthroscopic anterior cruciate ligament reconstruction: a randomized controlled trial

**DOI:** 10.1186/s12871-026-04109-y

**Published:** 2026-07-22

**Authors:** Funda Atar, Fatma Özkan Sipahioğlu, Savaş Altınsoy, Atakan Sezgi, Yusuf Özgüner, Özge Yamankılıç Mumcu, Derya Özkan, Jülide Ergil

**Affiliations:** Department of Anesthesiology and Reanimation, University of Health Sciences, Etlik City Hospital, Ankara, Türkiye

**Keywords:** Anterior cruciate ligament reconstruction, Ambulatory surgical procedures, Nerve block, Pain management, Analgesia

## Abstract

**Objectives:**

The aim of this study was to compare the 4-in-1 block technique with femoral nerve block (FNB) and adductor canal block (ACB) in terms of perioperative pain and opioid consumption in patients undergoing arthroscopic anterior cruciate ligament reconstruction (ACLR).

**Methods:**

The trial was retrospectively registered. In this randomized controlled trial, patients scheduled for ACLR under general anesthesia were randomly assigned to one of three groups: 4-in-1 block, FNB, or ACB. All blocks were performed under ultrasound guidance with 20 mL of 0.25% bupivacaine approximately 45 min before surgery. Intraoperative remifentanil use, postoperative 24-hour intravenous morphine equivalents (IME) consumption, and pain scores measured by the numerical rating scale (NRS) were recorded. Overall postoperative pain burden was additionally assessed using the 0–24-hour NRS area under the curve (AUC). An exploratory post-hoc non-inferiority analysis was performed.

**Results:**

A total of 60 patients were equally divided among the groups. Intraoperative remifentanil consumption, postoperative 24-hour IME consumption, and the 0–24-hour NRS AUC values did not differ significantly among the groups (*p* = 0.842, *p* = 0.502, and *p* = 0.958, respectively). Compared with the ACB group, the 4-in-1 block group used, on average, 0.8 mg less postoperative 24-hour IME (90% confidence interval [CI], − 5.61–4.01). Compared with the FNB group, 24-hour IME use was 2.8 mg greater (90% CI, − 1.48–7.08). In both comparisons, the upper bounds of the two-sided 90% CIs did not exceed the exploratory post-hoc non-inferiority margin (Δ = 10 mg).

**Conclusion:**

In patients undergoing ACLR, the 4-in-1 block did not differ significantly from FNB or ACB in terms of analgesic outcomes. These exploratory findings require further investigation to clarify the role of the 4-in-1 block in perioperative pain management for ACLR.

**Trial registration:**

ClinicalTrials.gov identifier: NCT05643040. Registered 23 November 2022. Retrospectively registered.

**Supplementary Information:**

The online version contains supplementary material available at https://doi.org/10.1186/s12871-026-04109-y.

## Introduction

Arthroscopic anterior cruciate ligament reconstruction (ACLR) is a commonly performed surgery to restore functional stability to the knee. However, severe postoperative pain can hinder early rehabilitation, increase morbidity, and negatively impact quality of life [1, 2]. Multimodal analgesia has become the standard approach for pain management after ACLR, and regional nerve blocks are a key component of this approach. To date, no clear consensus exists regarding the most effective regional anesthesia technique for knee surgery. Femoral nerve block (FNB), which is commonly applied during these procedures, provides reliable pain relief but is not a purely sensory block [1]. The adductor canal block (ACB) has been described as an alternative technique based on its anatomical location and predominantly sensory target profile [3]. Although these nerve blocks usually suffice for pain control following ACLR, certain surgical interventions—such as hamstring graft harvesting, posterior tibial fixation, or repairs involving the posterior capsule or meniscal horn—may require additional nerve block techniques [4]. However, the need for additional or multiple block techniques may increase procedural complexity, require higher local anesthetic doses, and complicate perioperative management [5]. Therefore, regardless of the surgical technique used, an effective and practical technique capable of blocking large areas of innervation around the knee is needed.

The 4-in-1 block is an ultrasound-guided peripheral nerve block technique designed to target a broad analgesic area around the knee and below-knee region [5, 6]. The term “4-in-1” refers to the concept that a single injection administered at the adductor canal–hiatus region aims to simultaneously affect four nerve branches contributing to the sensory innervation of the knee (the saphenous nerve, obturator nerve, popliteal plexus, and the branch to the vastus medialis nerve). The primary aim of this technique is to provide more comprehensive analgesia in situations where single-nerve-focused approaches, such as FNB or ACB, may be insufficient, without the need for additional block procedures [7]. Accordingly, the 4-in-1 block has been proposed as an alternative to classical multimodal block combinations requiring multiple injections; however, clinical experience with this technique remains limited.

Previous studies of the 4-in-1 block have predominantly focused on postoperative analgesic efficacy [5–7]. In contrast, the effect of this technique on intraoperative opioid requirements in comparison with other block approaches has been less extensively evaluated. Therefore, remifentanil consumption measured under standardized anesthetic management and predefined titration conditions was selected as the primary outcome to quantitatively compare intraoperative opioid requirements among the block techniques.

## Materials and methods

### Trial registration and protocol deviations

The study was approved by the local institutional ethics committee (6 June 2022; Ref: 139/13) and was registered at ClinicalTrials.gov (23 November 2022; NCT05643040). Patient recruitment began before trial registration, with the first participant enrolled on 1 September 2022, and enrollment was ongoing at the time of registration.

Although the trial was registered using the broad term “knee surgery,” the study was planned from the outset to include only patients undergoing arthroscopic ACLR. This scope was defined before participant enrollment and was not amended during recruitment or after trial initiation. Patient screening and eligibility assessment were restricted to individuals scheduled for arthroscopic ACLR. Therefore, no patients undergoing other types of knee surgery were considered for inclusion. Accordingly, all enrolled participants underwent ACLR, and the cohort presented in this manuscript represents the entire study population rather than a selected subgroup analysis. Written informed consent was obtained from all enrolled participants.

### Inclusion and exclusion criteria

Patients ≥ 18 years with American Society of Anesthesiologists (ASA) I–III scheduled for elective unilateral arthroscopic ACLR under general anesthesia were eligible. Patients who declined participation, had coagulopathy, peripheral neuropathy, allergy to local anesthetics, chronic opioid use, anticoagulant use, or pregnancy were excluded.

### Anesthesia protocol

During the preadmission clinic visit, the research coordinator met with eligible patients and provided information about the nerve block procedures, the numerical rating scale (NRS) (0–10; 0: no pain, 10: worst imaginable pain), and the intravenous patient-controlled analgesia (PCA) (CADD-Legacy PCA Pump, Smiths Medical International, Inc., St. Paul, MN 55112, USA) program. Demographic data, routine laboratory tests, and medical history were recorded, and a physical examination was performed.

### Study interventions

In this prospective, randomized, exploratory study, three nerve block techniques were compared. Patients were randomly allocated to three groups—4-in-1 block, FNB, and ACB—in a 1:1:1 ratio via a computer-generated random number program. The randomization sequence was generated by an independent statistician who was not involved in the study, and each allocation was placed into opaque, sequentially numbered, and sealed envelopes. Patients, surgeons, ward and operating room nurses, outcome assessors, and the researchers performing the statistical analyses were kept unaware of group allocation from the outset of the study.

The blocks were administered approximately 45 min before surgery in the preoperative preparation room. This timing was selected to allow sufficient sensory block development, particularly for the 4-in-1 block, and to ensure that all blocks reached an adequate clinical effect before surgery [7]. Sensory evaluation was performed according to the expected anatomical distribution of each block: the anterior thigh and anterior knee for the FNB, the medial knee for the ACB, and the anterior and posterior aspects of the knee for the 4-in-1 block. Block success was defined as a clear reduction or complete loss of pinprick sensation compared with the contralateral side before surgical incision. All assessments were performed by blinded independent investigators using the same predefined sensory criteria. Block onset time was not formally measured, and motor block was not assessed.

Intravenous access was established for all patients, and standard monitoring was applied. Patients were placed in the supine position with the operative-side hip externally rotated and the knee flexed. Skin anesthesia was achieved via the use of 1–2 mL of 1% lidocaine at the planned needle insertion site. All blocks were performed under aseptic conditions and ultrasound guidance (HFL 38X/13–6 MHz; SonoSite M-Turbo ultrasound system; SonoSite Inc., Bothell, WA, USA) via a 22-G, 10 cm long stimulating needle (Echoplex, Vygon, Ecouen, France) and 20 mL of 0.25% bupivacaine. All randomized patients received the allocated intervention and were included in the intention-to-treat analysis. As no exclusions occurred after randomization, the per-protocol and intention-to-treat populations were identical.

#### Adductor canal block

To accurately determine the injection level for the ACB, the transition between the femoral triangle and the adductor canal was identified using ultrasound guidance. The sartorius muscle was traced proximally and distally along the thigh to define this transition. The apex of the femoral triangle was defined as the point where the medial border of the sartorius muscle intersects with the medial border of the adductor longus muscle. Injections performed distal to this anatomical landmark were considered to be within the adductor canal. Although the saphenous nerve could not always be clearly visualized, it was typically identified lateral to the femoral artery. During block performance, the needle was advanced in-plane through the sartorius muscle and directed into the adductor canal immediately lateral to the femoral artery. The needle trajectory was adjusted from anterolateral to posteromedial, and the local anesthetic was injected adjacent to the femoral artery within the canal [8].

#### Femoral nerve block

The ultrasound probe was positioned at the inguinal crease to identify the femoral artery, vein, nerve, and iliopsoas muscle. The femoral nerve was identified as a hyperechoic structure beneath the fascia iliaca, lateral to the artery and above the iliopsoas muscle. Using an in-plane approach, the needle was advanced laterally to medially toward the femoral nerve, and local anesthetics was administered.

#### 4-in-1 block

**Fig. 1:**
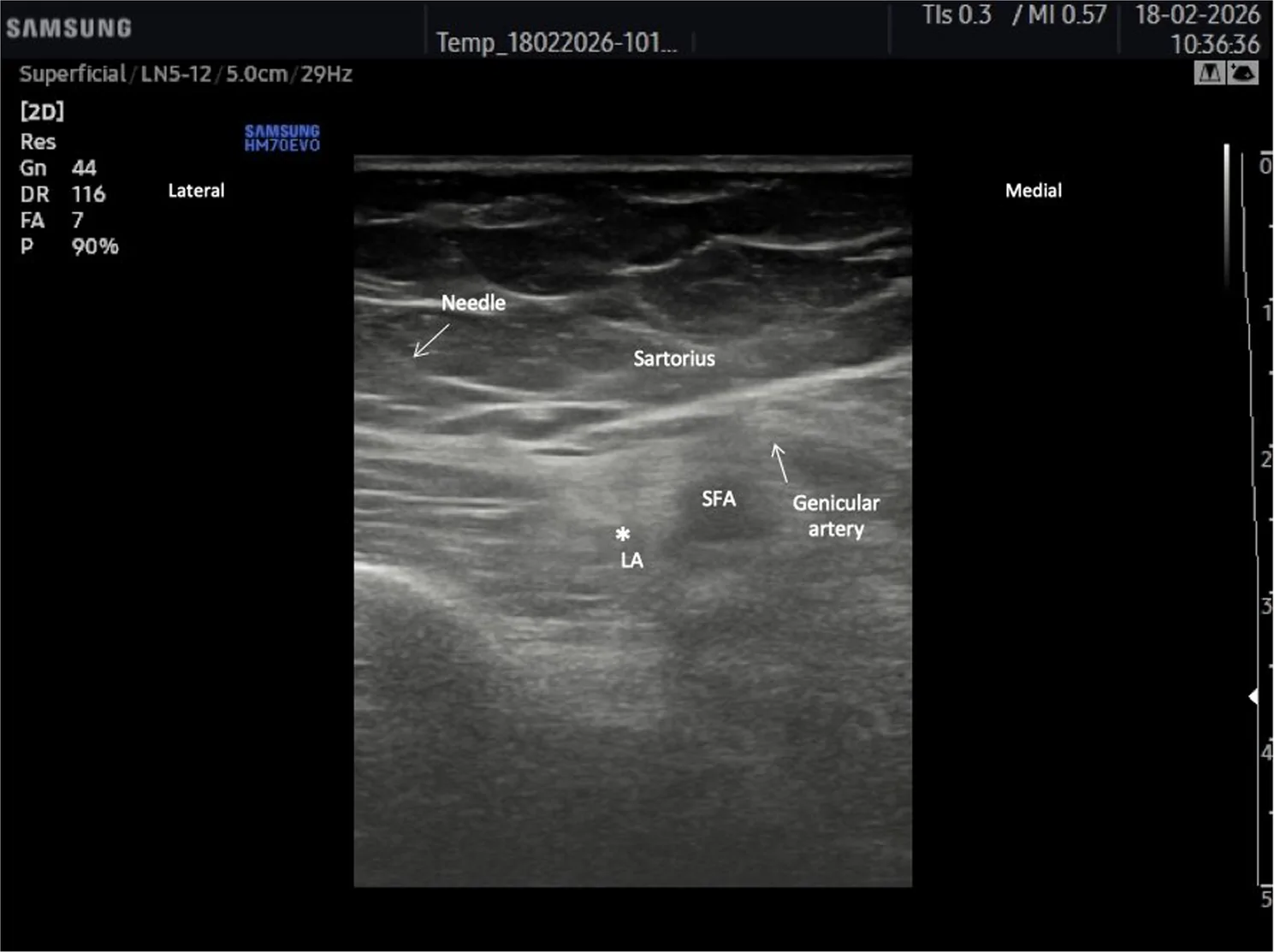
Ultrasound-guided injection point for the 4-in-1 block. SFA: Superficial femoral artery, LA: Local anesthetic, *: needle tip

A high-frequency linear ultrasound probe was placed over the medial femoral condyle. The junction between the vastus medialis and sartorius muscles, known as the anteromedial intermuscular septum, was identified. The probe was then moved proximally to visualize the superficial femoral artery and the descending genicular artery. The needle was advanced in-plane from lateral to medial toward a point approximately 8–10 cm proximal to the femoral condyle. After confirming negative aspiration, saline hydrodissection was performed, followed by injection of the local anesthetic, and its spread around the artery was observed. Under ultrasound guidance, the spread of the local anesthetic toward the adductor hiatus and the popliteal plexus was qualitatively assessed (Fig. 1).

### Intraoperative and postoperative management

Preoxygenation was performed with 100% O₂ with tidal-volume ventilation for 3 min. Induction was performed with intravenous propofol at 2 mg/kg. A neuromuscular blocker was not administered. Ventilation was maintained with a laryngeal mask to achieve an end-tidal carbon dioxide level of 35–40 mmHg. The propofol infusion rate was adjusted to maintain a bispectral index (BIS) between 40 and 60. Remifentanil (Ultiva^®^, GlaxoSmithKline, Germany) was administered as a continuous infusion within a dosage range of 0.2–0.5 µg/kg/min, titrated according to hemodynamic responses. Intraoperative hypotension (systolic blood pressure decrease > 20% from baseline) was managed by decreasing the remifentanil infusion rate by 0.05 µg/kg/min and administering fluid replacement. In patients with intraoperative hypertension (systolic blood pressure increase > 20% from baseline), the remifentanil infusion rate was increased by 0.05 µg/kg/min. If hypertension persisted after reaching the maximum remifentanil dose, initiation of an intravenous nitroglycerin infusion and titration according to hemodynamic response were planned (0.3–1.0 µg/kg/min). Bradycardia (heart rate < 50 bpm or > 20% decrease from baseline) was treated with 0.01 mg/kg atropine (repeated if necessary). All patients received 8 mg of intravenous dexamethasone and 4 mg of ondansetron. ACLR procedures were performed by the same surgical team following similar surgical steps. Patients were transferred to the postanesthesia care unit (PACU) after they met the recovery criteria.

All patients received the same standardized non-opioid multimodal analgesic regimen. At the end of surgery, before emergence from anesthesia, the first dose of intravenous paracetamol (1 g) was administered to all patients. During the postoperative period, patients received 1 g of intravenous paracetamol every 6 h. Oral diclofenac was initiated at the time of first oral intake and administered at a dose of 50 mg every 8 h. No deviations from this protocol occurred. PCA was set to deliver 20 mg tramadol (Contramal^®^) bolus doses with a 20-minute lockout interval; no background infusion was administered. Postoperative pain was assessed via the NRS during standardized lower-limb movement (assisted knee flexion–extension) while patients remained in bed in the PACU and at postoperative hours 1, 4, 8, and 24. Total tramadol consumption in the first 24 postoperative hours was recorded and calculated via conversion to intravenous morphine equivalents (IME). This conversion was performed to express postoperative opioid consumption in a standardized metric and to facilitate comparison across patients and with previous studies. Tramadol was converted to IME using a parenteral potency ratio of 10:1 (i.e., 100 mg intravenous tramadol corresponds to 10 mg intravenous morphine), based on published pharmacological data [9]. IME calculations included only postoperative PCA-administered tramadol, and no additional rescue opioids were used. Intraoperative remifentanil consumption was reported as total dose (µg), and an additional sensitivity analysis normalized to surgical duration was performed. Opioid-related side effects (itching, urinary retention, nausea, and vomiting) were also recorded. Potential complications related to regional anesthesia, such as nerve injury and hematoma, were evaluated by routine clinical observation for neurological symptoms during the early postoperative period and throughout the in-hospital stay; no structured post-discharge neurological follow-up was performed.

The primary outcome was intraoperative remifentanil consumption, used to assess intraoperative opioid requirements. The secondary outcome was postoperative analgesia efficacy, assessed by 24-hour postoperative opioid consumption expressed as IME. Postoperative NRS scores were recorded at predefined time points as supportive pain assessments.

### Statistical analyses

Statistical analyses were conducted using IBM SPSS 22.0 (IBM Corporation, Armonk, NY, USA). Normality of data distribution was assessed using the Kolmogorov–Smirnov test. Normally distributed continuous variables are presented as mean ± standard deviation, whereas non-normally distributed variables are expressed as median and interquartile range (IQR). Categorical variables are presented as numbers and percentages (%). Postoperative pain over time was summarized by calculating the area under the NRS–time curve (AUC) for each patient using the trapezoidal rule to reflect the overall pain burden. Between-group comparisons were performed using one-way ANOVA, the Kruskal–Wallis test, or the Pearson chi-square test, as appropriate. A p value < 0.05 was considered statistically significant.

Although the study was originally designed to assess superiority, an exploratory post-hoc non-inferiority analysis was additionally performed to evaluate whether clinically unacceptable differences could be excluded. For this exploratory post-hoc non-inferiority analysis, mean differences (4-in-1 − comparator) were calculated using independent samples t-tests, and two-sided 90% confidence intervals (CIs) were constructed. The non-inferiority margin of + 10 mg for IME consumption was selected based on clinical reasoning and prior literature reporting a median minimal clinically important difference (MCID) of 10 mg IME for 0–24 h postoperative opioid consumption [10–12]. For this exploratory analysis, the upper bound of the two-sided 90% CI was compared with the + 10 mg non-inferiority margin. No formal a priori sample size calculation was performed.

## Results

**Fig. 2:**
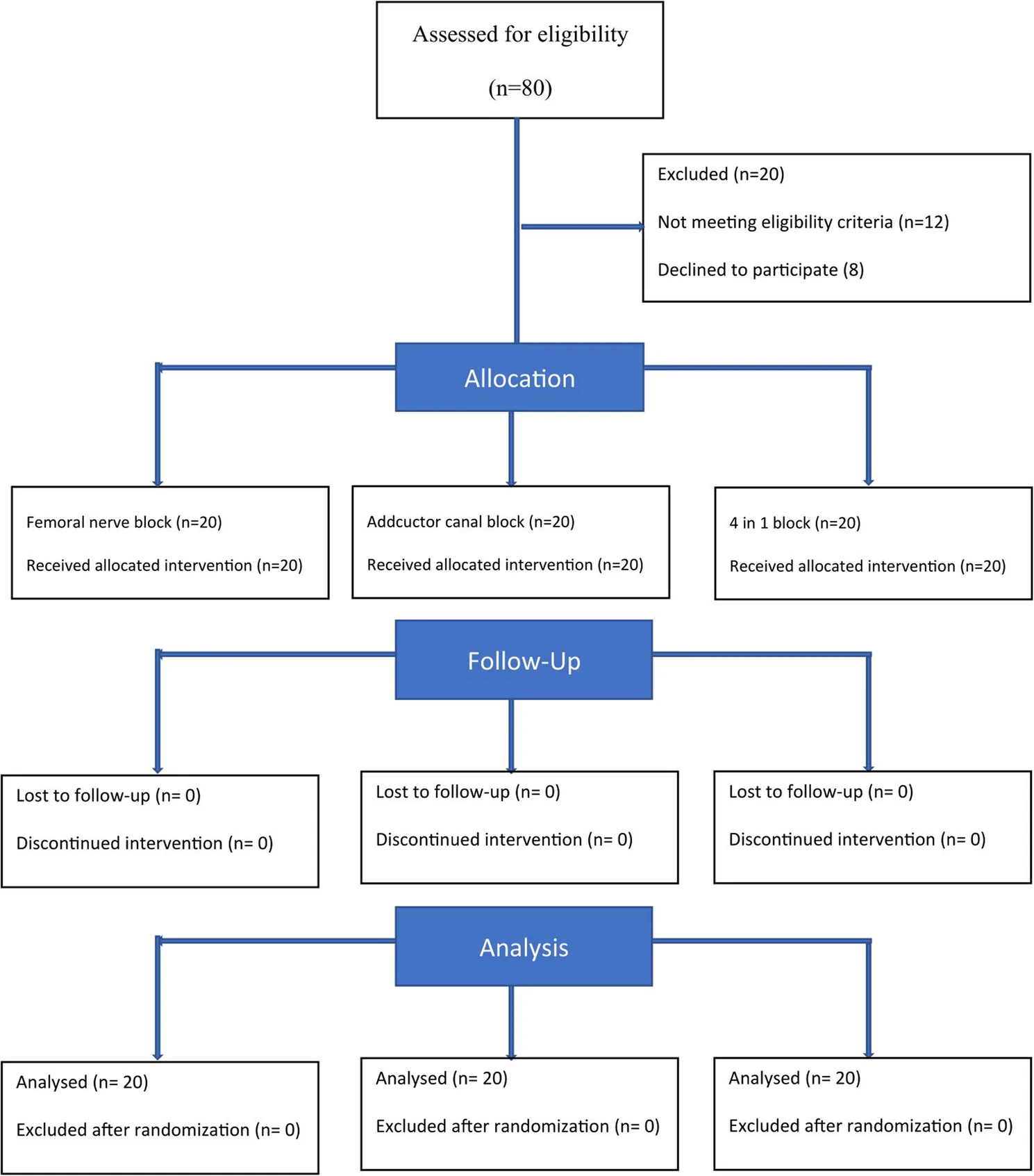
Flow diagram of patient recruitment

The flowchart of patient selection is illustrated in Fig. 2. To evaluate postoperative pain following ACLR, 60 patients were randomly assigned to the FNB (*n* = 20), ACB (*n* = 20), and 4-in-1 block (*n* = 20) groups. No failed or clinically incomplete blocks requiring rescue regional anesthesia or conversion to an alternative block technique occurred in any study group. Accordingly, all randomized patients were included in the final analysis, and the intention-to-treat and per-protocol populations were identical. Among the enrolled patients, 43 (71.7%) were male, with a mean age of 31.8 ± 10.9 years (range: 18–57) and a mean body mass index (BMI) of 27.4 ± 4.2 kg/m² (range: 20.2–38.1). The mean duration of surgery was 80.2 ± 23.5 min (range: 40–132). There were no statistically significant differences among the groups with respect to baseline demographic characteristics or duration of surgery (all p values > 0.05) (Table 1).

**Table 1:** Demographic characteristics and duration of surgery in patients receiving adductor canal block, femoral nerve block, or 4-in-1 block

	Adductor canal block (*n* = 20)	Femoral nerve block (*n* = 20)	4-in-1 block (*n* = 20)	*p* value
Age (years)	31.2 ± 10.2	32.5 ± 9.6	30.1 ± 8.6	0.736*
Male, n (%)	13 (65)	15 (75)	15 (75)	0.720^β^
BMI, (kg/m^2^)	26.8 ± 4.8	27.8 ± 3.7	27.7 ± 4.2	0.693*
ASA (I/II), n	13/7	13/7	12/8	0.931 ^β^
Duration of surgery (min)	76.5 ± 27.0	74.3 ± 23.1	89.8 ± 17.3	0.077*

Values are presented as the mean ± SD (standard deviation) or n. *BMI* body mass index, *ASA* American Society of Anesthesiologists. *: One-way ANOVA; β: Chi-squared test

Intraoperative remifentanil consumption, expressed both as total dose and as µg/min, was similar across groups (*p* = 0.842 and *p* = 0.656, respectively). Mean ± SD postoperative IME values were 19.2 ± 9.7 mg [95% CI: 14.64 to 23.76] in the ACB group, 15.6 ± 6.0 mg [95% CI: 12.81 to 18.39] in the FNB group, and 18.4 ± 13.3 mg [95% CI: 12.18 to 24.62] in the 4-in-1 group, with no significant differences observed among the groups (*p* = 0.502).

NRS scores measured in the PACU and at postoperative hours 1, 4, 8, and 24 were comparable among groups (*p* = 0.703, *p* = 0.769, *p* = 0.925, *p* = 0.984, and *p* = 0.842, respectively). Consistent with these findings, the 0–24-hour NRS AUC values did not differ significantly among groups (*p* = 0.958). Mean ± SD AUC values were 97.3 ± 44.9 [95% CI: 76.30 to 118.34] in the ACB group, 93.3 ± 46.5 [95% CI: 71.57 to 115.12] in the FNB group, and 96.1 ± 41.2 [95% CI: 76.85 to 115.49] in the 4-in-1 group (Table 2).

**Table 2:** Postoperative pain scores and opioid consumption in patients receiving adductor canal block, femoral nerve block, or 4-in-1 block

	Adductor canal block (*n* = 20)	Femoral nerve block (*n* = 20)	4-in-1 block (*n* = 20)	*p* value
Total remifentanil consumption(µg, median [IQR])	350.00 [626.00]	200 [715.75]	279.00 [411.00]	0.842^α^
Remifentanil consumption (µg/min, median [IQR])	0.07 [0.10]	0.04 [0.13]	0.04 [0.09]	0.656^α^
Postoperative IME consumption (mg, mean ± SD)	19.2 ± 9.7	15.6 ± 6.0	18.4 ± 13.3	0.502*
NRS (mean ± SD)				
PACU	3.9 ± 2.7	3.5 ± 3.4	4.3 ± 2.9	0.703*
1. h	4.7 ± 2.4	4.4 ± 2.4	5.0 ± 2.4	0.769*
4. h	4.5 ± 2.6	4.2 ± 2.2	4.3 ± 2.4	0.925*
8. h	4.1 ± 2.0	4.2 ± 2.2	4.2 ± 1.8	0.984*
24. h	3.8 ± 2.3	3.4 ± 2.2	3.5 ± 2.2	0.842*
AUC (0–24 h)	97.3 ± 44.9	93.3 ± 46.5	96.1 ± 41.2	0.958*

Normalized values were calculated by dividing total remifentanil consumption by surgical duration (minutes). Values are presented as the mean ± SD or median [IQR]. *SD* standard deviation, *IQR* interquartile ranges, *IME* intravenous morphine equivalent, *NRS* Numeric Rating Scale, *PACU* Postanesthesia care unit, *AUC* Area under the curve*: One-way ANOVA; α: Kruskal–Wallis test

**Fig. 3:**
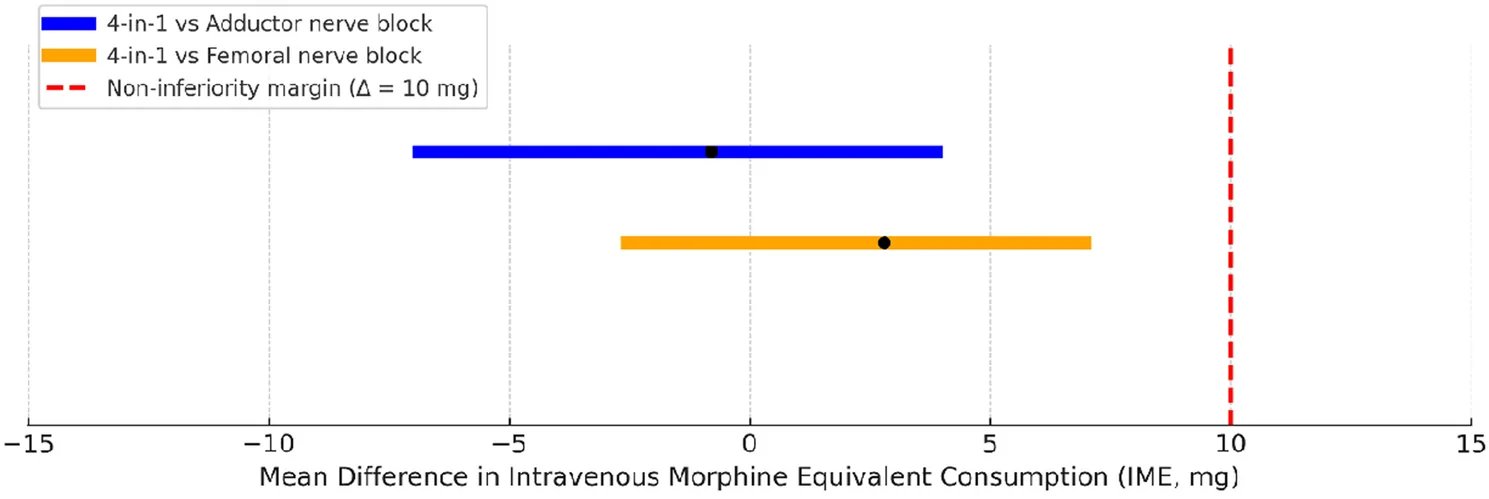
Exploratory post-hoc non-inferiority plot displaying the mean difference in intravenous morphine equivalent (IME) consumption between the 4-in-1 nerve block and the adductor canal block or the femoral nerve block. The black dots indicate mean differences; the horizontal bars represent two-sided 90% confidence intervals (CIs). The dashed red line marks the non-inferiority margin (Δ = 10 mg). In this exploratory post-hoc analysis, the upper limit of the CI did not exceed the margin

In an exploratory post-hoc non-inferiority analysis, postoperative 24-hour IME was on average − 0.8 mg (90% CI: −5.61–4.01) lower in the 4-in-1 block group than in the ACB group and 2.8 mg (90% CI: −1.48 to 7.08) higher than in the FNB group. In both comparisons, the upper bound of the two-sided 90% CI did not exceed the non-inferiority margin of + 10 mg (Fig. 3).

No early in-hospital complications related to regional analgesia techniques, such as nerve injury or hematoma, were observed in any of the groups. All patients were discharged on the first postoperative day.

## Discussion

The 4-in-1 block is a relatively novel peripheral nerve block technique that has been described for analgesia in knee surgery. In this study, no significant differences were observed in postoperative analgesic outcomes between the 4-in-1 block and FNB or ACB in patients who underwent ACLR under general anesthesia.

The complex innervation of the knee complicates the selection of the optimal peripheral nerve block. The anteromedial region of the knee is mainly innervated by the genicular branches of the saphenous nerve and the nerve to vastus medialis; the posterior region is innervated via the posterior branch of the obturator nerve and the popliteal plexus [13]. This anatomical variability leads to differences in the neural structures targeted by various block techniques and, consequently, in their potential areas of effect. FNB and ACB are commonly used techniques to provide analgesia around the knee; however, the extent of their analgesic coverage may vary depending on the level of injection and the spread pattern of the local anesthetic. In particular, in ACB, the level at which the injection is performed is a critical factor that determines the spread of the local anesthetic and, consequently, the clinical mechanism of action. In the ACB, administering the injection at a more proximal or distal level may influence the spread of the local anesthetic toward the femoral triangle or the adductor hiatus, as well as its relationship with the popliteal plexus [14]. In this study, ACB was performed distal to the apex of the femoral triangle, that is, within the adductor canal, and no significant difference was observed in postoperative NRS scores or opioid consumption when compared with FNB. The 4-in-1 block aims to target a broader sensory area with a single injection by affecting anteromedial knee sensation via the saphenous nerve and the nerve to the vastus medialis, and, through distal spread, the popliteal plexus and the posterior branches of the obturator nerve [5]. In our study, although the spread of the local anesthetic toward the adductor hiatus and the popliteal plexus was qualitatively observed under ultrasound guidance, no separate assessment of posterior region–specific pain was performed. Therefore, the findings should be interpreted within the context of the overall analgesic effect around the knee. Differences in injection level and local anesthetic spread across overlapping neural structures should be considered when interpreting the clinical effects of different block techniques.

In patients undergoing ACLR, postoperative analgesic outcomes did not differ significantly between the 4-in-1 block and either FNB or ACB. By contrast, Salem et al. demonstrated that the use of the 4-in-1 block after total knee arthroplasty resulted in lower postoperative pain scores and decreased opioid requirements compared with FNB and ACB [15]. This observed analgesic advantage may be related to procedural differences between total knee arthroplasty and arthroscopic ACLR, particularly with respect to surgical invasiveness. In ACLR, postoperative pain exhibits a more heterogeneous pattern shaped by the distribution of surgical portal entries, graft harvesting, and soft tissue manipulation considered together [16]. Moreover, the relatively high volume of local anesthetic used in the study by Salem et al. may have contributed to the observed analgesic superiority. In our study, identical doses and volumes of local anesthetic were administered across all groups, and a standardized multimodal analgesia protocol was applied in addition to the nerve blocks. Under multimodal analgesia protocols, the translation of an additional increase in sensory coverage into clinical pain scores may have remained limited due to a “ceiling effect” [17]. Taken together, these factors suggest that the 4-in-1 block may not have demonstrated a clear analgesic superiority over ACB and FNB in our study. The exploratory post hoc non-inferiority analysis should be interpreted with caution, as the study was not designed or powered to establish non-inferiority.

The absence of a significant difference among the groups in the present study should not be interpreted as indicating that these three techniques provided an absolutely high level of analgesia in ACLR. Indeed, the persistence of moderate postoperative NRS values over time and the continued opioid requirement in all groups suggest that a residual pain burden remained despite multimodal analgesia. This observation is also consistent with previous studies reporting that ACB and FNB provide similar pain control after ACLR, but do not completely eliminate postoperative analgesic requirements [3, 18]. Harbell et al. likewise reported that, despite the combined use of a more comprehensive regional technique involving femoral and sciatic nerve blocks, postoperative pain was not completely abolished, with the median highest PACU pain score remaining at 5 and reaching as high as 10 in some patients [19]. These findings may be explained by the multicomponent nature of pain after ACLR. In ACLR, postoperative pain may arise not only from intra-articular surgical trauma, but also from graft harvesting, portal entry sites, bone tunnels/fixation, and manipulation of the surrounding soft tissues [19, 20]. This residual pain may also have been influenced by the technical and pharmacological characteristics of the analgesic strategy. Differences in injection level, local anesthetic volume, and spread may have affected the extent of neural coverage [21, 22]. In addition, the composition of the multimodal analgesic regimen may alter postoperative opioid requirements, suggesting that the specific regimen used in this study may also have contributed to the residual pain burden [23]. Therefore, in this surgical setting, postoperative pain scores may not be sufficiently robust to distinguish the specific effect of a single block technique. Nevertheless, because our study did not include an unblocked control group, these findings should not be interpreted as indicating that the techniques were generally ineffective. Our findings suggest that there was no clear separation among the 4-in-1 block, FNB, and ACB with respect to postoperative analgesic outcomes.

This study has several limitations. First, no objective assessment of quadriceps strength, motor function, or postoperative mobilization was performed. Accordingly, comparisons between block techniques were limited to analgesic outcomes, and no conclusions can be drawn regarding Enhanced Recovery After Surgery (ERAS)-related early mobilization or motor recovery. The postoperative follow-up of the patients was limited to the duration of hospital stay. Therefore, outcomes and adverse effects occurring after discharge could not be assessed. Our results are specific to the local anesthetic dosages used, and it should be noted that different dosages may yield different outcomes. Tramadol-to-IME conversion ratios may vary across the literature. Pain assessment was based on patient-reported outcomes, inherently introducing subjectivity into the data. Although intraoperative remifentanil consumption was assessed under standardized anesthetic management and predefined titration criteria, it may still be influenced by multiple clinical and perioperative factors. Surgical technical details such as graft type, fixation technique, and concomitant procedures (e.g., meniscal repair or chondral interventions) were not systematically recorded. These factors can significantly influence postoperative pain and opioid requirements. The trial was not designed or powered to establish non-inferiority; therefore, the post-hoc non-inferiority analysis should be regarded as exploratory. A formal a priori sample size calculation was not performed, and the sample size was based on feasibility and expected patient volume during the study period. Therefore, the study may have had limited statistical power to detect small between-group differences, and the possibility of a type II error should be considered when interpreting the study findings. The exact outcome definitions, assessment time points, and detailed analytical methods reported in the manuscript were not fully prespecified in the trial registration or documented in a separate prospectively dated protocol or statistical analysis plan. Finally, although this was a prospective randomized study, the trial was retrospectively registered after enrollment had already started, and the registry described the study outcomes only in broad terms rather than with the exact operational definitions reported in the manuscript.

## Conclusion

In patients undergoing ACLR, no significant differences in analgesic outcomes were observed between the 4-in-1 block and FNB or ACB. The findings are exploratory, and further studies are needed to better define the role of the 4-in-1 block in perioperative pain management for ACLR.

## Supplementary Information

Supplementary Material 1.

## Data Availability

The datasets generated and/or analyzed during the current study are available from the corresponding author on reasonable request.

## Abbreviations

ACLR: Anterior cruciate ligament reconstruction
FNB: Femoral nerve block
ACB: Adductor canal block
ASA: American Society of Anesthesiologists
BMI: Body mass index
NRS: Numerical rating scale
PCA: Patient-controlled analgesia
BIS: Bispectral index
PACU: Postanesthesia care unit
IME: Intravenous morphine equivalent
MCID: Minimal clinically important difference
AUC: Area under the curve
CI: Confidence interval
ERAS: Enhanced Recovery After Surgery
